# Analysis of medical malpractice liability disputes related to novel antineoplastic drugs and research on risk prevention and control strategies

**DOI:** 10.1371/journal.pone.0286623

**Published:** 2023-06-05

**Authors:** Jinyu Luo, Zaoqian Zheng, Rongliang Yu

**Affiliations:** 1 Division of Nursing, Hemopurification Center, Tongde Hospital of Zhejiang Province, Hangzhou, Zhejiang Province, People’s Republic of China; 2 Department of Pharmacy, Tongde Hospital of Zhejiang Province, Hangzhou, Zhejiang, People’s Republic of China; 3 Division of Medical Administration, Tongde Hospital of Zhejiang Province, Hangzhou, Zhejiang, People’s Republic of China; 4 Division of Medical Administration, Zhejiang Academy of Traditional Chinese Medicine, Hangzhou, Zhejiang, People’s Republic of China; University of Foggia: Universita degli Studi di Foggia, ITALY

## Abstract

**Objective:**

To investigate the general characteristics of litigation cases of medical malpractice liability disputes (MMLDs) related to novel antineoplastic drugs (NADs), the drugs involved, as well as the common types of medical errors related to NADs and their damages in the process of diagnosis and treatment, with the aims of improving the level of rational medication use in the clinical application of NADs and actively prevent medical disputes.

**Methods:**

The China Judgments Online was searched for the cause of action using the key word “MMLDs” along with the name of 77 kinds of NADs. A total of 39 NAD litigation cases meeting the inclusion criteria from 1 January 2009 to 31 December 2021 were analyzed, and each potential adverse drug reaction (ADR) was reviewed to determine a causality assessment using the Naranjo algorithm for non-drug-induced liver injury (DILI) cases and the updated Roussel Uclaf Causality Assessment Method (RUCAM) for the DILI cases. Risk prevention and control strategies were recommended.

**Results:**

Cases that met the inclusion criteria increased substantially each year during the last six years, from three cases in 2009–2015 to 36 cases in 2016–2021. There were more cases in Eastern China than in other geographic regions. Most cases involved tertiary hospitals, patients between 25 and 60 years of age, and patients who were predominately male. There were 18 kinds of NADs involved in medical errors. The most common consequences of NADs were closely related to the death, disability, and increased treatment costs caused by ADRs, inadequate indications, delayed diagnosis and treatment, and misdiagnosis and mistreatment. The most frequent medical errors **were** medical technology errors, medical ethics errors and medical record writing/safekeeping errors. In two cases involving DILI, one case was unable to undergo further RUCAM scoring because the liver function indicators of the patient before and after treatment were not published.

**Conclusion:**

The establishment of mechanisms to reduce the risks associated with the clinical application of NADs is warranted. Healthcare services must maintain strict adherence to the specific requirements of *GPCANADs* and drug instructions and strictly grasp the indications, contraindications, usage, and dosage of drugs, and strengthen the notification and management of off-label drug use. Monitoring patients for ADRs and preparing rescue and treatment measures for high-risk drugs may serve to reduce damages related to NADs. For DILI cases, medical and appraisal institutions should use RUCAM score to assess causal relationships.

## Introduction

Novel antineoplastic drugs (NADs) refer to small molecule-targeted drugs and macromolecular monoclonal antibodies, which are commonly used to treat cancer [1]. Cancer is the leading cause of death in China and developed countries [2], and has become a major public health problem in China [3]. The number of cancer cases and deaths, as well as crude incidence and mortality of cancer in China, have increased gradually since 2000 [4], and there will be an estimated 4,820,000 new cases of cancer, and 3,210,000 cancer-related deaths in China in 2022 [5]. Based on the current treatment of malignant tumors, traditional chemotherapy drugs can no longer fully meet the clinical needs [6]. Targeting NADs at specific sites can reduce the side effects to some extent and significantly improve the mortality and poor prognosis of tumor patients [5]. In 2018, the National Health Commission of China issued the first edition of the *Guiding Principles for the Clinical Application of NADs* (hereafter referred to as the *GPCANADs*) [7], which has been updated each year and has now been updated to the fifth edition (2022) [8]. From the 2018 to 2022 Edition, many new kinds of NADs included in GPCANADs increased year by year, reaching 34, 46, 60, 77 and 103, respectively [7–11]. In addition, the National Health Commission has attached great importance to the clinical application of antitumor drugs and has taken a series of actions to improve cancer diagnosis and reasonable drug use in China. It has successively issued clinical application management norms such as measures for the clinical application of antitumor drugs (Trial) [12], management indicators for the rational clinical application of antitumor drugs (2021 Edition) [13], and an action plan for improving the quality of cancer diagnosis and treatment [14].

The high incidence of NAD-related toxic and side effects and with the improvement of the availability of NADs, their use has become increasingly widespread, and the rationality of their clinical application and the risk of medical-legal disputes have aroused widespread concern [15,16]. Medical malpractice liability (MML), or tort liability, typically leads to compensation for medical damages. MML commonly results from damages caused by failures in diagnosis and treatment activities due to medical errors. Medical errors refer to the negligence of healthcare services and personnel who fail to fulfill the necessary duty of care in diagnosis and treatment. Tort cases resulting from medical errors are commonly referred to as medical malpractice liability disputes (MMLDs) [17]. They are a serious public health problem of common concern and a leading cause of death worldwide [18].

The Identification of medical errors is the key component in the trial of medical tort cases. Legal norms, judicial interpretation, and diagnosis and treatment norms together constitute the foundation for the identification of medical errors [19]. It is challenging to uncover a consistent cause of these errors. If found, it may prove difficult to provide a consistent and viable solution that minimizes the chances of reoccurrence. However, by recognizing and learning from these occurrences, improvements can be made to prevention efforts and increased patient safety. An assessment of MMLDs associated with NAD-related litigation has not previously been reported. This type of research is crucial for improving knowledge and understanding of the risks associated with NADs, to educate medical service providers on strategies to reduce risks to patients, and to establish mechanisms for risk prevention and control within management of healthcare systems. This study was designed as an investigation of medical errors and MMLDs linked to the use of NADs in China to provide a substantive basis for the development of risk prevention and control strategies.

## Materials and methods

### Data sourcing and search strategy

The China Judgments Online is a unified platform provided by the Supreme People’s Court of China for the publication of judicial documents from all Courts across the Country [20]. Litigation cases are published on this platform after judgement. This platform has been used previously to analyze MMLD litigations throughout China [21]. All information used in this study was obtained from publicly available court records and no confidential or protected health records were obtained. Therefore, no approval from an Institutional Review Board was required, and the requirement for informed consent was waived. Participants’ identities were not required during or after data collection.

On April 30, 2022, two investigators extracted litigation cases with the judgment date between 1 January 2009 to 31 December 2021 from China Judgments Online and a combination of MMLDs (as cause of action) and the name of 77 different kinds of NADs included in the *GPCANADs* (2021 Edition) [11], was used for the key word search (Table 1).

**Table 1:** Small molecule targeted drugs and macromolecular monoclonal antibody drugs included in the *GPCANADs*.

Primary targets [22]	Drug Name and other targets	Kinds
**Small molecule inhibitors** [23]		**45**
ALK TKIs	Alectinib (RET); Ceritinib (RY); Crizotinib* (C-MET/ROS1); Ensartinib	4
BCL-2 inhibitors	Venetoclax	1
BCR-Abl inhibitors	Dasatinib (PDGFR); Nilotinib; Imatinib* (C-KIT, NRY)	3
BRAF inhibitors	Dabrafenib; Vemurafenib	2
BTKI	Ibrutinib (NRY); Zanubrutinib	2
EGFR TKIs:	Afatinib* (ErbB1/2/4, RY); Almonertinib; Dacomitinib; Erlotinib* (RY); Furmonertinib; Gefitinib*; Icotinib; Neratinib (ErbB2/HER2); Osimertinib	9
JAK/ STAT	Ruxolitinib	1
MEK inhibitors	Trametinib	1
MET TKIs	Savolitinib	1
pan- HER2 TKIs	Pyrotinib	1
PARP inhibitor	Fluzoparib; Niraparib; Olaparib; Pamiparib	4
RET inhibitors	Pralsetinib	1
FLT3 /AXL inhibitors	Gilteritinib	1
Multi-targeting TKIs	Anlotinib (VEGFR/FGFR/PDGFR/KIT); Apatinib* (VEGFR2/STAT3/PD-L1 /ROS/Nrf2/p62); Avapritinib (PDGFRA/KIT); Axitinib (VEGFR/PDGFR/KIT); Donafenib; Fruquintinib (VEGFR); Lapatinib (EGFR/ErbB2/HER2); Lenvatinib (VEGFR/FGFR/PDGFR/KIT/RET); Pezopanib* (VEGFR/PDGFR/FGDR/KIT); Sorafenib* (VEGFR/PDGFR/C-RAF/B-RAF); Sunitinib (VEGFR/PDGFR/KIT/RET); Surufatinib (VEGFR/FGFR1/CSF-1R); Regorafenib (VEGFR/PDGFR/RET/RAF); Ripretinib (KIT/PDGFR)	14
**Proteasome inhibitors**		**2**
Proteasome inhibitors	Bortezomib*; Ixazomib	2
**Monoclonal antibodies (mAbs)** [24]		**10**
Human/murine, chimeric anti-CD20	Rituximab*	1
BiTEs CD19/CD3	Blinatumomab	1
CD38	Daratumumab	1
EGFR	Cetuximab; Nimotuzumab*	2
HER2	Pertuzumab; Inetetamab; Trastuzumab*	3
RANKL	Denosumab	1
VEGF-A targeting mAbs	Bevacizumab*	1
**Antibody-drug conjugates (ADCs)** [25]		**3**
CD30	Brentuximab Vedotin	1
HER2	Disitamab Vedotin; Trastuzumab Emtansine	2
**Immunotherapy**		**13**
**Immune checkpoint inhibitor (ICIs)**		**9**
PD-L1	Atezolizumab; Durvalumab	2
PD-1	Camrelizumab; Nivolumab*; Pembrolizumab; Sintilimab*; Tislelizumab; Toripalimab*	6
CTLA-4	Ipilimumab	1
IMiD	Lenalidomide; Pomalidomide; Thalidomide	3
mTOR inhibitors	Everolimus*	1
**Others**		**4**
Inhibiting tumor angiogenesis	Endostatin	1
HDAC inhibitors	Chidamide*	1
CDK4/6 inhibitors	Abemaciclib; Palbociclib	2
**Total**		**77**

**Abbreviations:** ALK = Anaplastic lymphoma kinase; TKIs = Tyrosine kinase inhibitors; RET = Rearranged during transfection; RY = Receptor protein-tyrosine kinase; C-MET = Cellular mesenchymal-epithelial transition; ROS = Reactive oxygen species; BCL = B-cell lymphoma; PDGFR = Platelet-derived growth factor receptor; NRY = Non-receptor protein-tyrosine kinase; BRAF = V-raf murine sarcoma viral oncogene homolog B1; BTKI = Bruton’s tyrosine kinase inhibitors; EGFR = Epidermal growth factor receptor; HER = Human epidermal growth factor receptor; JAK = Janus kinases; STAT = Signal transducer and activator of transcription; MET = Mesenchymal epithelial transition factor; PARP = Poly(ADP-ribose) polymerase; FLT = FMS-like tyrosine kinase; VEGFR = vascular endothelial growth factor receptor; FGFR = fibroblast growth factor receptor; PD = Programmed cell death; L = ligand; CSF = Colony-stimulating factor; BiTEs = Bispecific T cell engager; RANKL = receptor activator of NF-κB ligand; CTLA = Cytotoxic T lymphocyte-associated antigen; IMiD = Immunomodulatory agent; mTOR = Mammalian target of rapamycin inhibitors; HDAC = Histone deacetylase;

* = Drugs involved in the litigation cases.

### Inclusion and exclusion criteria

Cases were considered eligible for inclusion if they met the following criteria: 1) Cases centered on consequences of MMLDs with NADs listed as medical errors, 2) Judgments included full-text information, and 3) Only the record of the final judgment was kept. If judgment data were incomplete, additional data were obtained by consulting corresponding records from the same case.

Cases deemed irrelevant were excluded. These included cases that were retrieved through the keyword search, but context was not centered on consequences of NADs or duplicate judgment documents of the same case were retrieved. If one case was reported in multiple records, the duplicate was eliminated. Both investigators reviewed the full text of each record to further analyze the judgments and to determine if each document met the inclusion and exclusion criteria. A third investigator was consulted if consensus was not reached.

### Data extraction

Data extraction was carried out using pre-designed forms. Data were extracted by one researcher and then thoroughly checked by a second researcher. Data extraction included: 1) Basic information about the judgment, 2) Patient demographics and clinical characteristics, 3) Basic information about the disputes, 4) Judgment on MMLDs in the record, and 5) Analysis of medical errors related to the dispute. In addition, a search through national medical institutions [26] and the hospitals official websites to query the level of medical institutions was performed. All costs have been expressed in US dollars (USD). Based on the average exchange rate in 2021, 1 USD was equivalent to approximately 6.4515 Chinese Yuan (CNY).

### Definitions

Disability degree identified by judicial appraisal institution refers to the degree of the victim’s loss of ability to work. Disability degree is divided into levels 1–10 Level. 1 is the highest degree of disability while Level 10 is the lowest [27].

Responsibility of the medical service provider as judged by the court refers to the tort liability that medical institutions or their medical personnel must accept. In addition, it indicated the main form of compensation for damages due to medical errors in the medical process. This also includes cases of legal provisions with or without medical errors.

### Causality assessment of ADRs

Each potential ADR was reviewed independently by two investigators to determine a causality assessment using the Naranjo algorithm [28] for the non-DILI cases and the updated Roussel Uclaf Causality Assessment Method (RUCAM) [29] for the DILI cases. Previously, each potential ADR case had been analyzed by a judicial authentication institution and the causal relationship between NADs and ADRs was confirmed. However, outside RUCAM, the details of assessment were usually not presented in DILI case reports, making reassessment by investigators difficult, and therefore the Naranjo algorithm was also used for DILI cases. The total scores and resulting causality grades are reported in Table 2.

**Table 2:** Total score and resulting causality grading.

Naranjo algorithm		Roussel Uclaf Causality Assessment Method	
Score	Causality grading	Score	Causality grading
≤ 0	doubtful	≤ 0	excluded
/	/	1–2	unlikely
1–4	possible	3–5	possible
5–8	probable	6–8	probable
≥9	definite	≥9	highly probable

### Statistical analysis

Descriptive analysis was performed using GraphPad Prism software version 8.0 (GraphPad Prism Software, Inc., San Diego, CA, USA). Continuous data were reported as mean ± standard deviation (SD). Categorical data were presented as number and percentage (%).

## Results

### Literature retrieval and study characteristics

**Fig 1:**
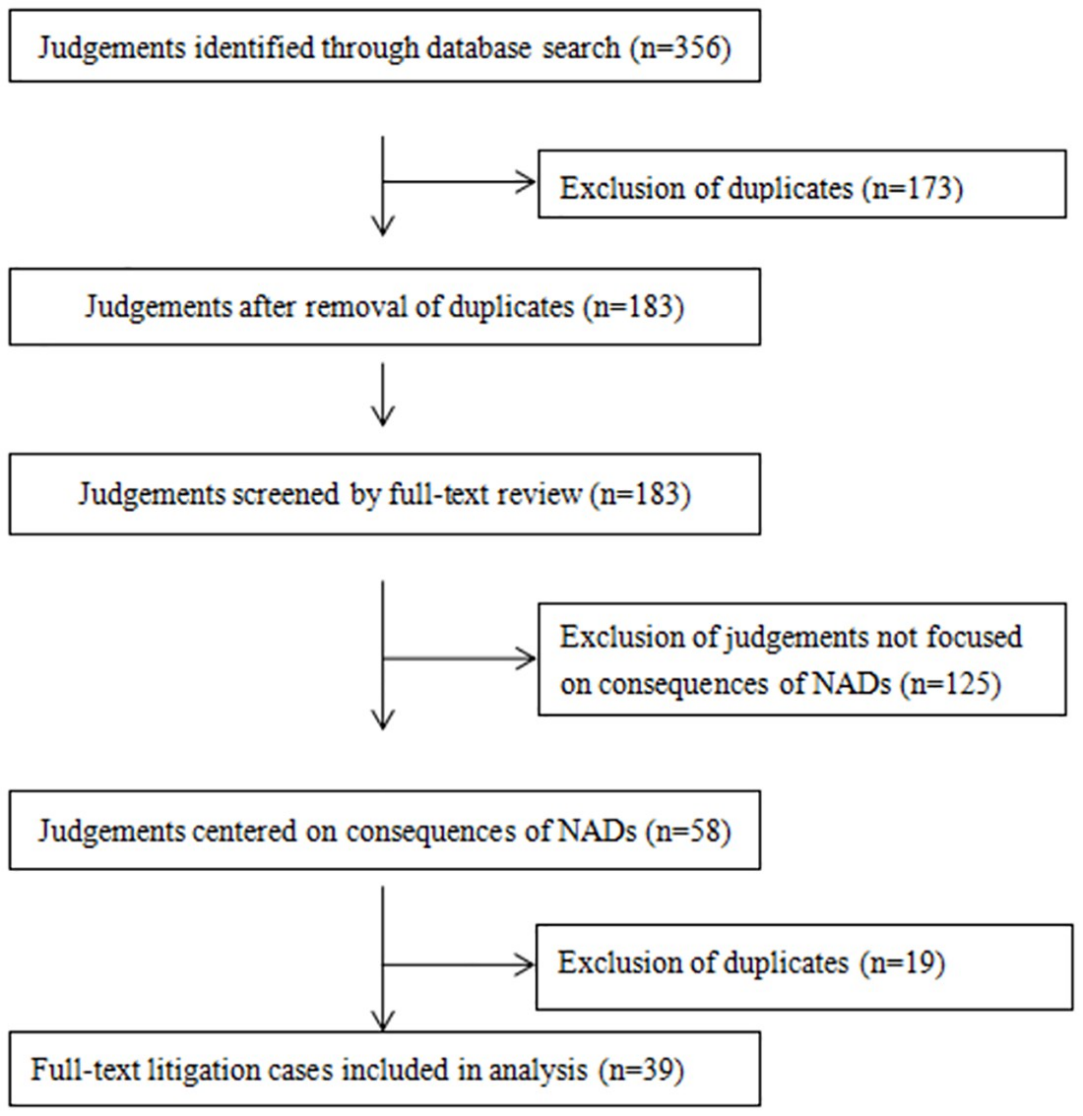
Flowchart showing study selection process.

The search strategy yielded a total of 356 judgments, of which 173 were excluded after removal of duplicates. Another 125 judgments that did not fall within the scope of the study based on full-text assessment were excluded. Of the remaining 58 judgments, 39 cases met all the inclusion criteria. The judgment selection flow diagram is shown in Fig 1. The literature retrieval and study characteristics are presented in S1 Table.

### General characteristics of included NAD-related judgments

The general characteristics of the 39 cases in the scope of this research are detailed in Table 3. Twenty-three cases were completed in the court of first instance, accounting for 58.97% of the cases. The remaining sixteen cases proceeded to the second instance or retrial and execution process of the cases. The original judgment was upheld for 13 of these cases (81.25%).

**Table 3:** General characteristics of medical malpractice liability cases involving NADs (N = 39).

Characteristics	No. of cases	Percent (%)
Progress of lawsuit		
First instance	23	58.97
Second instance	11	28.21
Retrial and execution process	5	12.82
Uphold the original judgment	13	81.25
First instance register date		
2009–2015	3	7.69
2016–2021	36	92.31
Region		
East China	17	43.59
Central China	8	20.51
North China	8	20.51
Northeast China	1	2.56
Northwest China	0	0
Southwest China	1	2.56
South China	4	10.26
Type of medical institution		
Three-level hospital/Class A	37/36	94.87/92.31
Two-level hospital	2	5.13
Type of identification institution		
Medical association	10	23.81
Judicial expertise institution	32	76.19
Gender		
Male	25	64.10
Female	14	35.90
Age		
25≤age≤60	23	58.97
60<age≤75	7	17.95
age˃75	7	17.95
Unmentioned	2	5.13
Patient outcome at judgment		
Death cases	29	74.26
Disability	5	12.82
Others	5	12.82
Autopsy of death cases	2	6.90
Appraisal fee ($)	Mean	SD
Patient	1,139	1,091
Hospital	949	806
Total	2,088	1,111
Court acceptance fee ($)	Mean	SD
Patient	523	641
Hospital	767	955
Total	1,290	1,224

Note: Some cases registered in 2021 may still be under trial, and there is no judgment at present, or the judgment has not been uploaded to the China Judgements Online database.

Cases that met the inclusion criteria increased substantially each year during the previous six years, from three cases in 2009–2015 to 36 cases in 2016–2021. Over 43% of cases were from Eastern China, more than any other geographic region. Thirty-seven cases (36 Class A) were from tertiary hospitals while the other two cases were from secondary hospitals. The number of cases identified by judicial expertise institutions was higher than those identified by medical associations, accounting for 76.19%. Patient ages ranged from 25 to 90 years. For cases where the judgment included data for age and sex, 58.97% of patients were between 25 and 60 years old, and 64.10% of patients were male. Twenty-nine patients died at the time of judgment. Autopsies were performed for only two of these patients. The average total cost of the appraisal fee was $2,088. Patients and hospitals were responsible for $1,139 and $949 of these fees, respectively. The average total cost of the court acceptance fee was $1,290, of which patients and hospitals were responsible for $523 and $767, respectively. The detailed summary of thirty-nine-cases concerning NADs are presented in S2 Table.

### Drugs and NAD-related damage payments

Eighteen kinds of NADs were involved in medical errors, accounting for 23.38% of the 77 kinds of NADs listed in the *GPCANADs* (2021 edition). However, 16 kinds of NADs involved in medical errors were included in the 2018 edition, which accounted for almost half (47.06%) of the NADs listed in this edition. The average propotion of responsibility attributed to medical service providers regarding the damages was 34%. The most frequently used NADs involved in medical errors were rituximab, bortezomib, apatinib, and imatinib (Table 4). The most common damages involving NADs were death, disability, and increased treatment costs caused by ADRs, insufficient indications (II), delayed diagnosis and treatment (DDAT), and misdiagnosis and mistreatment (MAM). There were 19 deaths, two cases of disability, and one case of additional expenses related to ADRs (total of 22 cases, accounting for 56.41% of all cases). There were six deaths and one case of disability related to II (total of seven cases, accounting for 17.95% of all cases). There were two deaths, one case of disability, and four cases of additional expenses related to MAM (total of seven cases, accounting for 17.95% of all cases). There were two deaths and one case of disability related to DDAT (total of three cases, accounting for 7.69% of all cases). The average compensation for damages was $34,589 per case with the highest judgement of $97,301. The detailed NADs associated with medical errors and the causal relationship between errors and damages are detailed in Table 4.

**Table 4:** Drugs and NAD-related damages.

Drugs (n = 18)	Case number	Disease	Main issues	Medical errors#	Causality assessment of ADRs				Damages	Responsibility (%)	Compensation ($)	No. (%)
					Type of ADRs	Scores	Grading	Method				
Rituximab	1	Lymphoma	ADRs	1①abc;1②bd;1③ab;4①;8⑨	Cardiogenic shock (Infusion-related reactions)	6	probable	N	Death	50	76,722	5 (12.82)
	2	Systemic lupus erythematosus; Fahrenheit macroglobulinemia	ADRs	1①gh;3⑤;4①;5①	Acute pulmonary edema	6	probable	N	Death	25	40,253	
	3	Hodgkin’s lymphoma	ADRs	1①h;5②;7①e;7②ab;8③	Drug-induced lung injury (Interstitial pneumonia)	3	possible	N	Death	25	58,607	
	4	Anti-neutrophil cytoplasmic antibody (ANCA) associated vasculitis	ADRs	1②a	Severe pneumonia	3	possible	N	Death	10	10,287	
	5	Non-Hodgkin’s lymphoma	ADRs	1①bcg;3④; 7①ae	Hepatitis B virus (HBV) reactivation	3	possible	N	Disability (grade 7)	90	96,889*	
Imatinib	6	Gastric stromal tumor	ADRs	1①bcde	Hepatitis B virus (HBV) reactivation	6	probable	N	Death	25	44,581	4 (10.26)
	7	Acute lymphoblastic leukemia (Ph+)	DDAT	6①a	/	/	/	/	Death	10	23,435	
	8	Gastrointestinal stromal tumor with low malignant potential	DDAT	6①b;7①ac	/	/	/	/	Disability (grade 7)	70	43,821	
	9	Mesenchymal tumors with low malignant potential	MAM	4①;4②a;5③	/	/	/	/	Disability (grade 7)	15	21,841	
Sorafenib	10	Renal malignant tumor	ADRs	1②c;1③c;7①ce;8①;8②	Drug-induced liver injury	U	/	/	Expenses	20	870	1 (2.56)
Afatinib	11	Lung adenocarcinoma	ADRs	1③d	Drug-induced liver injury	6	probable	R	Death	15	10,587	1 (2.56)
Toripalimab	12	Esophageal cancer and lung cancer	ADRs	1①g;1②d;7⑧	Immunotherapy related side effects	3	possible	N	Death	10	5,869	1 (2.56)
Nivolumab	13	Lung cancer	ADRs	1②c;7⑤	Sudden cardiac death	3	possible	N	Death	15	53,388	1 (2.56)
Bortezomib	14	Multiple myeloma	ADRs	2①	Transient acute ADRs (blood pressure decline, thrombocytopenia)/Peripheral neuropathy	6/3	Probable/possible	N	Disability	60	75,582	5 (12.82)
	15	Multiple myeloma	ADRs	1①g;1②c;1③e;2①;7①bdf;7④;8③	Acute lung injury (ALI)/Acute respiratory distress syndrome (ARDS)	6	probable	N	Death	65	52,100	
	16	Multiple myeloma	ADRs	1①i;1②a;1③i;7①g;8⑧	Severe pneumonia, ARDS	3	possible	N	Death	40	73,761	
	17	Multiple myeloma	ADRs	1③h;2②	Cardiac failure	3	possible	N	Death	20	12,500	
	18	Multiple myeloma	ADRs	1②a;7⑥	Cardiac failure	2	possible	N	Death	U	12,400	
Apatinib	19	Lung squamous cell carcinoma	ADRs	1③f;2③a;7④	Massive hemoptysis	6	probable	N	Death	20	15,954	4 (10.26)
	20	Lung adenocarcinoma	ADRs	7①f;8③;8④	Massive hemoptysis	3	possible	N	Death	30	61,249	
	21	Gastric cancer	ADRs	1③f;2③b;7⑤;8③	Gastrointestinal bleeding	3	possible	N	Death	15	18,847	
	22	Cervical cancer	ADRs	1③g;2③a;5①	Gastrointestinal bleeding	3	possible	N	Death	35	51,476	
Bevacizumab	23	Brain glioma	ADRs	1①f;1③bce;2③a;3④;6①e;8⑨	Gastrointestinal bleeding	6	probable	N	Death	60	87,874	3 (7.69)
	24	Liver cancer	ADRs	1①g;2③a;7①f;7④;7⑦;8④	Gastrointestinal bleeding	3	possible	N	Death	10	20,540	
	25	Diffuse astrocytoma	II	2③a;7④	/	/	/	/	Disability (grade 5)	30	43,214	
Crizotinib	26	Lung cancer	ADRs	3④;7①e	Pulmonary embolism	3	possible	N	Death	0	1,550	2 (5.13)
	27	Primary bronchial lung cancer	II	3④;4②b	/	/	/	/	Death/Expenses	60	25,446	
Pezopanib	28	Carcinoma of renal pelvis	II	2③a;7④	/	/	/	/	Death	70	10,000	1 (2.56)
Chidamide	29	Non-Hodgkin lymphoma	MAM	6③	/	/	/	/	Death	10	7,750	1 (2.56)
Gefitinib	30	Undifferentiated large cell lung cancer	II	1③c;2③a;3③;8⑤;8⑦	/	/	/	/	Death	0	0	3 (7.69)
	31	Lung cancer	II	3⑤;6②;7①f;7③;8③;8⑥	/	/	/	/	Death	5	8,957	
	32	Lung cancer	II	4①	/	/	/	/	Death	10	6,227	
Nimotuzumab	33	Nasopharyngeal carcinoma	II	4①;3①; 7①adef	/	/	/	/	Death/Expenses	35	26,166	2 (5.13)
	34	Nasopharyngeal carcinoma	MAM	5①	/	/	/	/	Expenses	80	30,243	
Trastuzumab	35	Breast cancer (cT1N0M0)	MAM	2③;3①;3②;4②c;8④	/	/	/	/	Expenses	70	13,248	
	36	Breast cancer	DDAT	6①c;7①c	/	/	/	/	Death	15	13,299	2 (5.13)
Erlotinib	37	Hodgkin lymphoma	MAM	4①;5①	/	/	/	/	Expenses	100	97,301	1 (2.56)
Everolimus	38	Breast cancer	MAM	5③;6①d	/	/	/	/	Death	50	92,959	1 (2.56)
Sintilimab	39	Liver cancer	MAM	5①	/	/	/	/	Expenses	20	3,168	1 (2.56)
Mean±SD										34±27	34,589±29,871	39 (100)

Note: Causation in medical malpractice liability disputes is especially complex [30]. There are many causes and one consequence of medical damage. Medical damages are the result of the joint action of many factors such as patient’s own disease progression and negligent diagnosis and treatment behavior, inherent risk of medical behavior. Some factors not related to drugs are not listed.

Abbreviations: ADR = Adverse drug reaction; N = Naranjo causality assessment method; R = Roussel Uclaf Causality Assessment Method; II = Insufficient indications; DDAT = Delayed diagnosis and treatment; MAM = Misdiagnosis and mistreatment; U = Unable to judge;

* = The right to claim follow-up treatment is reserved.

^#^Medical errors (please refer to Table 5).

### Types of NAD-related medical errors

We found a total of 136 medical errors, with an average of 3.49 errors per case. The most frequent errors were medical technology errors, followed by medical ethics errors and medical record writing / safekeeping errors, which accounted for 63.97%, 24.26%, and 11.76% of the total medical errors, respectively. Among the medical technology errors, the management of ADRs ranked first, followed by the use of off-label drugs, which accounted for 30.15% and 8.82% of the total medical errors, respectively. The different categories of NAD-related medical errors are shown in Table 5.

**Table 5:** Types of NAD-related medical errors.

Order number	Medical errors	No. (%)
**I**	**Medical technology errors**	**87 (63.97)**
**1**	**Errors in the management of NAD-related ADRs**	**41 (30.15)**
①	Inadequate preparation to prevent ADRs before using NADs.	18
a	Inadequate preparation of medical personnel prior to the infusion; the use of anti-allergic drugs was not prevented.	1
b	No quantitative examination of hepatitis B virus-DNA was conducted.	3
c	No preventive anti-hepatitis B virus treatment for hepatitis B virus carriers was conducted.	3
d	No consultation from the Department of Hepatology.	1
e	Failure to review liver function results.	1
f	Failure to control relevant indicators, such as blood pressure, within a normal range.	1
g	Insufficient awareness of ADRs for NADs, and inadequate assessment of patients with extremely high risk of ADRs.	5
h	Deficiencies in preventive measures, such as appropriate isolation and protection, attention to blood sugar monitoring and control, early detection of viruses, and use of appropriate drugs for persons particularly susceptible to secondary infections.	2
i	Not specified	1
②	Inadequate observation and monitoring	9
a	The patient has multiple high-risk factors for poor prognosis, and the medical service providers have not appropriately extended the observation time in hospital.	3
b	A lack of close observation during the first drug infusion.	1
c	When prodromal symptoms of ADRs occur, relevant examinations were not conducted, and ADRs were not detected in a timely manner, leading to worsening conditions. This includes failure to carefully monitor adverse cardiac reactions during treatment, such as not rechecking the myocardial zymogram after two sinus tachycardia.	3
d	Not specified	2
③	Inadequate disposal and treatment of ADRs	14
a	Failure to discontinue medication in a timely manner when ADRs are suspected.	1
b	Rescue facilities, measures, personnel, and processes were not standardized and there was a lack of contingency plans.	2
c	Failure to analyze and handle the patient’s condition and ADRs in a timely manner. For example, the patient with gastrointestinal bleeding who has not regained consciousness for a long time, and there may be other causes, but there is no sufficient analysis and differential diagnosis by relevant doctors.	3
d	Used the wrong dose to treat ADRs caused by NADs, leading to the discontinuation of NADs and tumor progression, such as doctors mistakenly writing a single dose of ondansetron 8 mg as 8 tablets.	1
e	Misdiagnosis and mistreatment of ADRs.	2
f	The patient already has abnormal coagulation function and other drugs that may increase the risk of bleeding was not stopped after using apatinib.	2
g	Hemorrhagic patients had slightly insufficient total blood transfusion volume and balance solution administration.	1
h	The critically ill patient was not accompanied by a doctor during transfer examination, or the doctor failed to arrive at the scene in time for rescue.	1
i	Not specified.	1
**2**	**Errors in off-label drug use**	**12 (8.82)**
①	Unapproved dose.	2
②	Unapproved route of administration.	1
③	Unapproved indication, such as: a. For other tumors; b. For early tumor.	9
**3**	**Errors in condition evaluation during diagnosis and treatment**	**10 (7.35)**
①	Treatment plan not determined through multidisciplinary consultation before using NADs.	2
②	Three physicians with different levels of authority failed to identify and correct incorrect records of genetic testing results.	1
③	Lack of efficacy monitoring and evaluation of patients with NADs.	1
④	Inadequate understanding, prevention, and evaluation of existing diseases.	4
⑤	Not specified.	2
**4**	**Errors in molecular target detection**	**9 (6.62)**
①	NADs requiring target detection were not tested or results were not obtained before using NADs.	6
②	The target detection results did not meet the target requirements for using NADs.	3
a	The c-KIT/PDGFRa gene mutation test results showed that the c-KIT gene in the sample was of wild type in exons 11, 9, 13, 17, and PDGFRa gene was of wild type in exons 12 and 18, such as imatinib.	1
b	Misunderstanding of the gene testing report, "RET fusion mutation, ALK exon deletion", without further gene testing to determine whether ALK fusion is positive, crizotinib was treated as ALK positive.	1
c	HER2 negative was wrongly recorded as positive, which led to the incorrect use of trastuzumab.	1
**5**	**Errors in histopathological diagnosis**	**8 (5.88)**
①	No histopathological examination was performed, or no histopathological results were obtained before using NADs.	5
②	No further histopathological biopsy evaluation was conducted for the possibility of tumor recurrence.	1
③	Lack of comprehensiveness and accuracy of immunohistochemical detection and pathological molecular typing diagnosis led to misdiagnosis as “three negative” breast cancer, leading to incomplete treatment plans and during the period; it was not determined that the patient’s breast cancer had high expression of estrogen receptor (ER), which is a hormone-dependent tumor.	2
**6**	**Inappropriate treatment of NADs**	**7 (5.15)**
①	Inappropriate timing of NAD administration.	5
a	Ph+ALL patients did not consider adding TKI (Imatinib) drugs in a timely manner.	1
b	No use of imatinib after surgery to reduce recurrence of gastrointestinal stromal tumors.	1
c	Treatment with trastuzumab was delayed due to high costs.	1
d	Lack of targeted anti-cancer therapy in patients with high expression of estrogen receptor (ER) in breast cancer.	1
e	Relevant indicators were not controlled within the scope of drug requirements.	1
②	Reuse medication that failed treatment;	1
③	Improper discontinuation of NADs, such as the doctor mistakenly diagnosed the patient as suffering from pancreatitis and stopped using chidamide.	1
**II**	**Medical ethics errors**	**33 (24.26)**
**7**	**Errors in insufficient disclosure of informed consent (Communication and notification)**	**33 (24.26)**
①	Before using NADs, medical service providers failed to fully inform the patient or his/her agent:	20
a	Patients of their condition.	3
b	The purpose of chemotherapy.	1
c	Drugs and efficacy.	3
d	Application method and dose.	2
e	Possible ADRs, risks and prognosis.	5
f	Detailed introduction to alternatives.	5
g	Not specified.	1
②	Changing the treatment plan:	2
a	Communication and notification in writing was not provided to the patient regarding the current treatment effect, the reasons for changing the treatment drugs, the advantages and disadvantages of the optional treatment plan, and the patient’s current condition.	1
b	Failure to inform the patient about alternative treatment options in the event of ineffective treatment.	1
③	Reusing ineffective NADs. The use of a drug that had previously failed, the necessity of using the drug again, and the probability of success and failure of the drug was not explained to the patient in detail.	1
④	Failure to fully explain the risks and benefits of off-label use.	5
⑤	Patients experienced ADRs or changes in their condition. After patient’s disease suddenly worsened and became critically ill, the medical service providers failed to communicate with their family members in time and inform them of their serious or critical condition in time.	2
⑥	No notification to perform autopsy.	1
⑦	The notification of special risks failed to fully inform the patients of the additional risks that may result from chemotherapy under the patient’s current special condition, which affected the patients and their families’ choice of treatment plan.	1
⑧	Not specified.	1
**III**	**Errors in medical record writing / safekeeping**	**16(11.76)**
**8**	**Does not meet the requirements of the “Basic Standards for Writing Medical Records”**	**16 (11.76)**
①	Outpatient medical records were not written during outpatient service.	1
②	No notices on drug use and suggestions for relevant inspection in outpatient medical records.	1
③	After chemotherapy, there were no progress notes or timely records, incomplete records, or no recorded changes in the patient’s condition were found.	5
④	The discharge summary was incomplete or lacked detail or error record, such as failure to inform the chemotherapy plan and medication precautions in writing or mistakenly informing the patient to perform targeted therapy.	3
⑤	There was a lack of accurate, scientific records of efficacy monitoring and evaluation in the process of diagnosis and treatment. This impacted the ability to clarify the treatment effect and formulate the next treatment plan.	1
⑥	The writing of rescue records was not standardized and lacked specific details.	1
⑦	There were inconsistent, erroneous records (the progress note was inconsistent with the actual situation), and modification with obvious copy and paste marks in the progress note.	1
⑧	Medical record archiving was not standardized, and medical records were mixed with those of other patients.	1
⑨	Not specified.	2
**Total**		**136(100)**

Note: Medical damage involves multiple causes and one consequence. The above table analyzes medical errors of NADs according to the appraisal analysis opinions and the court’s analysis opinions. Abbreviations: ADRs = Adverse drug reactions.

## Discussion

### Characteristics of litigation cases of NAD-related MMLDs

We found that 41.03% of the cases proceeded to the second or retrial and execution process, which is significantly lower than our previous research on severe cutaneous ADRs (55%) [21], which may be more acceptable to patients given the results of verdicts awarding payments for damages caused by NADs compared to the damage caused by severe cutaneous ADRs. However, medical service providers should still pay attention to the rational use of NADs. The original judgement for the majority (81.25%) of the cases was upheld in the second or retrial cases. However, the execution of the second instance or retrial increased the costs of litigation and delayed the dispute resolution. The average total appraisal fee and court acceptance fee are $2,088 and $1,290, respectively. Considering factors such as expenses associated with time and a lawyer for both hospital and patient, negotiations to deal with medical disputes may be a more cost-effective alternative.

Over the last six years, as compared with the previous six years, the number of NADs litigation cases has significantly increased year by year, from three cases in 2009–2015 to 36 cases in 2016–2021. One possible reason is that China has become one of the countries with the lowest affordable prices of NADs, and the accessibility, availability, and affordability of NAD have been significantly improved. The Chinese Government has taken a series of measures to ease the financial burden on patients with cancer and to reduce the price of costly cancer drugs. These include exempt tariffs on imported cancer drugs, initiation of centralized government negotiations and procurement of cancer drugs, and incorporation of more cancer drugs into the catalogue of medical insurance reimbursement [31]. The largest number of cases have been found in Eastern China (17 cases), as compared to no cases in Western China. This situation may be related to the fact that the economic and cultural level of Eastern China is higher than that of Western China, and patients in coastal areas of Eastern China are more likely to access NADs in terms of economic affordability after suffering from cancer. According to the data released by the National Health Commission, there were 3,275 tertiary hospitals(1,651 Class A), 10,848 secondary hospitals, 12,649 primary hospitals, and 9,798 non-classified hospitals in China in 2021 [32]. However, a total of 94.87% litigations of MMLDs related to NADs have occurred at tertiary hospitals (Class A 92.31%), which are the highest-level hospitals in China. While secondary hospitals only accounted for 5.13%. This may be related to the fact that tertiary hospitals are the preferred choice for cancer patients seeking the best treatment. Therefore, with the development of China’s social economy and the balanced development of China’s Eastern and Western economies, as well as the improvement of the application, accessibility, and availability of new NADs, medical service providers should pay more attention to the rational clinical application of NADs and take the initiative to prevent the risk of NADs.

In our study, 29 patients had died at the time of judgment, two of which underwent autopsy, accounting for only 6.90% of the death cases, which suggests that the autopsy rate of death cases is low. A relevant study [33] showed that the forensic autopsy results of 36 cases of medical disputes in a Class A tertiary hospital confirmed that the medical diagnosis of the cause of death was accurate in 23 cases, inaccurate in seven cases, and unclear in six cases. Therefore, autopsy plays a very important role in determining the cause of death of patients and exploring whether there were errors in medical behavior. However, because many MMLD patients have no objection to the doctor’s diagnosis and treatment during the medical process, the patient’s body may have been cremated or exceeded the recommended time limit for autopsy prescribed by law when the dispute occurs. In addition, due to the influence of traditional Chinese ideas, close relatives are unwilling to have an autopsy, so the autopsy rate of death dispute cases is very low. The exact cause of death can be determined through autopsy to comfort the deceased. It can also increase knowledge regarding the occurrence and development process of the disease and promote the progress of medical science. In cases without autopsy, clinical cause of death is usually analyzed by clinical experts and appraisers from identification institution according to the identification materials, but because the exact cause of death is unclear, it increases the uncertainty of determining the causal relationship between medical errors and damage consequences. Therefore, after the death of a patient, the medical service providers should carefully organize a discussion on the death cases to seek alternative causes. If necessary, they could organize a hospital wide discussion to determine the cause of death, to improve the accuracy of the determination of the cause of death. If the identification institution cannot infer the cause of death of the patient based on the appraisal materials, it will be returned to the court for judgment. *The Regulations on the Prevention and Handling of Medical Disputes (RPHMD)* and *The Civil Code of the People’s Republic of China* are important legal bases for resolving MMLDs. Among them, the provisions on "autopsy" clarify the rights and obligations between medical institution and patients. 1) The medical institution has the obligation to inform the close relatives of the deceased of the relevant provisions of the autopsy. 2) If doctors and close relatives of the deceased disagree regarding the cause of death of the patient, the close relatives of the deceased patient have the obligation to request the autopsy within the prescribed time. In our study, among the other 27 deaths without autopsy, the reason for death in 26 patients was inferred by the medical malpractice appraisal institution based on the identification materials, so this may reduce the impact on the outcome of the study. However, the cause of death for one sudden death patient could not be determined, making it impossible to assess the causal relationship between medical errors and damages. The court ruled that the adverse legal consequences should be borne by the party who failed to perform the autopsy obligation. The basic premise of the trial of medical dispute cases is to find out the facts and distinguish the responsibilities. Therefore, scientific publicity of the autopsy should be strengthened to improve the understanding of the autopsy. For MMLD cases involving death, autopsies can protect the legitimate rights and interests of both doctors and patients. Both doctors and patients should have a sense of prevention and evidence and for autopsies to be performed in a timely manner.

The gender ratio of male (64.10%) and female (35.90%) in NAD-related litigations is 1.79. The age-standardized rate of cancer mortality per 100,000 people in China in 2015 was 101.3 for male and 55.3 for female, with a gender ratio of 1.83 [4]. This shows that there is a certain correlation between the gender ratio of NAD-related litigations and the gender ratio of age-standardized rate of cancer mortality.

After the court accepts the lawsuit case of MMLDs in China, it will entrust the medical association or the judicial expertise institution with the duty of conducting appraisals to clarify the responsibility. In our study, we found that the proportion of entrusted judicial expertise institutions was 76.19%, significantly higher than that of medical associations (23.81%). The biggest difference between the two is that the appraisers of the judicial expertise institutions are composed of two or more appraisers of the institution. The appraisers are mainly forensic experts who can consult clinical experts on relevant issues during the appraisal process. The appraisers of the medical association are composed of more than three experts in the expert database, including clinical experts in relevant disciplines and one forensic expert. The identification procedures of the medical association can refer to the literature published by Hongzhi Lv et al. [34]. As of October 1, 2018, the implementation of *RPHMD* further clarified the identification status of the medical association. In the future, the proportion of entrusting the medical association to conduct identification may be further increased.

The high compensation for damages associated with the cases in our analyses indicated that NADs were associated with severe financial burden for medical service providers, patients, and society.

### Drugs, medical errors, and NAD-related damages

We divided medical errors into three categories and eight aspects.

#### Medical technology errors

There were six aspects of medical technology errors involved in this study. Errors involving ADRs control accounted for nearly half of the medical technology errors. Therefore, in the clinical application of NADs, the primary task is to control the ADRs of NADs. The study of litigation cases highlighted several errors in this scope. The causes of litigation are generally acute and serious ADRs to NADs. The most frequent errors were inadequate prevention and preparation, inadequate disposal and treatment, and inadequate observation and monitoring, accounting for 13.24%, 10.29%, and 6.62% of the total medical errors, respectively.

#### Errors in the management of NAD-related ADRs

*Rituximab*. All five cases were associated with serious ADRs to rituximab, including one infusion related reaction (IRR), three lung injuries and one hepatitis B virus reactivation (HBVr).

IRRs. Case 1. A 55-year-old male patient with lymphoma developed chest tightness and discomfort during the first infusion of rituximab for 100 minutes, but the delivery of the drug was not stopped. At 120 minutes, the patient’s discomfort worsened. Subsequently, the patient developed cardiac arrest and died of cardiogenic shock after rescue. The occurrence of IRRs, especially during the first infusion, is a main concern of rituximab [35]. The clinical manifestations of a rituximab-induced IRR typically occurs within a few minutes to 120 minutes after the start of infusion. Reactions may vary widely, from mild adverse events (AEs) to life-threatening reactions. Fatalities occurred in 0.04% to 0.07% of patients [36]. According to the *GPCANADs*, it is recommended to pay attention to the risk of an IRR during the use of brentuximab vedotin, inetetamab, ramucirumab, carfilzomib, daratumumab, camrelizumab, and penpulimab [8].

Based on the errors we identified and related literature, several measures are required to prevent and manage the occurrence of IRRs: 1) Special monitoring of patients with cardiac or pulmonary conditions [37]; 2) Systematic administration of prophylaxis to reduce the occurrence and severity of IRR. Anti-allergenic drugs should be used before each infusion of rituximab. If the treatment regimen used does not include glucocorticoids, glucocorticoids should also be used in advance; 3) Monitor the infusion speed during the first infusion [8] and evaluate and manage the patient’s discomfort in a timely manner, such as discontinuation of the offending drug; 4) Availability of anticipated prescriptions for nurses to begin urgent care prior to the arrival of the doctor; 5) All supportive care should be immediately available [38].

*Lung injury*. Case 2. A 35-year-old male patient with systemic lupus erythematosus / megaglobulinemia died of respiratory failure due to acute pulmonary edema following the fourth infusion of rituximab. Case 3. A 30-year-old male patient with Hodgkin’s lymphoma developed dyspnea and died on the 52nd day after starting rituximab, and was identified as being associated with respiratory failure caused by rituximab induced lung injury. Case 4. A 78-year-old female patient with anti-neutrophil cytoplasmic antibody associated vasculitis developed a fever within 24 hours after the second infusion of rituximab, resulting in respiratory failure due to severe pneumonia. At present, the discovery, diagnosis, and treatment of acute lung injury caused by rituximab chemotherapy remains a challenge [39]. Zheng D et al. [39] reported that the onset chemotherapy time was mainly distributed in two to four courses, the time between the onset of symptoms and the infusion of rituximab was eight to 49 days. Fever is the most common clinical manifestation of the disease, followed by cough and chest tightness [39]. Due to the lack of specificity of its early clinical symptoms, it often fails to attract people’s attention [39]. The severity of acute lung injury ranges from hypoxemia to respiratory failure. In severe cases, fatal lung disease can occur. According to its severity, different treatment methods need to be taken [40]. For patients with symptoms of acute lung injury, rituximab and chemotherapeutic drugs should be stopped immediately, and glucocorticoids should be used instead [41]. Patients with severe cases often require mechanically assisted ventilation [41]. Therefore, chest imaging should be performed and evaluated before using rituximab, especially in patients with existing pulmonary insufficiency or tumor lung infiltration. The patient should be observed for fever, cough, and other related premonitory symptoms during administration of the medication.

*HBVr*. Case 5. A 57-year-old male patient with non-Hodgkin’s lymphoma (B-cell lymphoma) who was hepatitis B surface antigen positive, experienced HBVr after the second infusion of rituximab. The use of rituximab enhanced the patient’s liver injury, cirrhosis, splenomegaly, and other sequelae, which constituted grade seven disabilities. The amount of compensation in this case ranks second among the 39 cases, and the number of lawsuits is at least more than 6 times. The medical institution was responsible for 90% of the liability for compensation and reserves the right of patients to follow-up litigation. This result is similar to findings in our previous studies on severe cutaneous ADRs [21]. Disability often leads to higher compensation levels, which may be related to the increased indirect costs caused by patient disability.

The manufacturers of rituximab added black box warnings to the product labels identifying rituximab as a high HBVr risk in 2013 [42]. HBVr is an overlooked but significant complication of common medical therapies that can delay treatment or result in clinical episodes of hepatitis, hepatic failure, or death [43]. HBVr is defined as a sudden and rapid increase in HBV DNA levels by at least 100-fold in those with previously detectable HBV DNA or a reappearance of HBV DNA viremia in individuals who did not have viremia prior to the initiation of immune suppressive or biological modifier therapy or cancer chemotherapy [44]. Prevention and treatment of HBVr is complex but is now a well-recognized and preventable complication in clinical practice. HBVr can be identified in a timely manner by combining ALT levels with HBV DNA and HBsAg. In HBsAg[+] patients, a dramatic rise in HBV DNA concentrations (usually 100-fold or more) are indicative of HBVr, while in patients with resolved HBV infection (HBsAg[−] and HBcAb[+]), HBVr usually means the reappearance of HBsAg or an increase in serum HBV DNA concentrations with or without HBsAg seroconversion and ALT exacerbation [45].

#### Imatinib

*HBVr*. Case 6. A male patient under 60 years old presented with a gastric stromal tumor had HBVr after imatinib treatment, which induced and promoted liver function injury. According to the *GPCANADs*, in addition to above drugs, it is also recommended to be aware of the risk of HBVr during the use of zanubrutinib, orelabrutinib, daratumumab, and Obinutuzumab [8].

Therefore, it is necessary to establish a comprehensive plan to manage the process when using NADs that have a risk of HBVr according to the errors mentioned in the aforementioned litigation case. These actions include: 1) Host-related, virus-related, and medication-related risk factors assessment. Patients with current or previous hepatitis B viral infections, the medical service providers should consult a hepatitis specialist before starting treatment and pay attention to monitoring during treatment; 2) Screening for viral hepatitis prior to initiation of NADs, such as conduct quantitative examination of hepatitis B virus DNA; 3) Monitoring liver function indicators; 4)Management of HBVr in individuals receiving immunosuppressive agents. The two strategies for targeting HBVr are antiviral prophylaxis and preemptive therapy. Antiviral prophylaxis means treating patients (usually at least 1 week before immunosuppressive therapy) with HBsAg[+] or HBcAb[+] regardless of viral load or whether or not there are clinical symptoms of HBVr. Preemptive therapy refers to the close surveillance of HBV DNA, in which antiviral therapy begins at the first sign of an increase in the HBV DNA load [45]; 5) Duration of therapy and monitoring. The optimal duration of prophylactic antiviral therapy remains controversial. Data derived from multiple sources indicate that antiviral prophylaxis should last for at least 6 months after the cessation of immunosuppressive therapy and should be lengthened to 12 months for patients receiving regimens with B-cell-depleting therapies or antiviral prophylaxis [45]. Due to space constraints, we will not be discussing the risk prevention and control strategies in detail and refer the readers to reviews on the topic [43–45].

The two HBVr cases cited above have been confirmed by judicial institutions as HBVr, but given that a large number of DILI cases are attributed erroneously to nondrug alternative causes, the lack of RUCAM evaluation results should not be disregarded [46]. We recommend conducting RUCAM assessments on patients with liver function injury. In these two patients, laboratory data covering liver function indicators were not disclosed in the court documents, which makes it difficult for us to draw any further conclusions.

In the other two cases, no hepatitis virus was detected, but the patients’ liver was seriously injured.

#### Sorafenib

*Drug-induced liver injury (DILI)*. Case 10. A male patient (age unknown) with a renal malignant tumor stage T3 had severe liver function injury during the use of sorafenib, which was diagnosed as DILI. After treatment, the patient’s liver function returned to normal. According to the appraisal opinion given by identification institution, the patient’s liver function damage was caused by sorafenib, and the DILI was not found in time, resulting in the aggravation of liver function injury. The healthcare services compensated 20% of the patient’s medical expenses, which included hospitalization food allowance, transportation, nursing, nutrition, lost wages, and appraisal fees, totaling $830. Unfortunately, this case occurred in 2015, and the liver function indicators of the patient before and after treatment had not been published, which made it impossible to conduct further RUCAM scoring.

#### Alfatinib

*DILI*. Case 11. A 79-year-old patient with advanced left lung adenocarcinoma (Phase IV B c (T2N3M1c) was diagnosed with DILI after taking alfatinib with a RUCAM score of 6 points. The patient was treated with ondansetron tablets due to ADRs of nausea and vomiting during oral afatinib treatment. The doctor mistakenly wrote the prescription for a single dose of 8 mg as 8 tablets, and the patient took 32 mg of ondansetron orally at one time, resulting in or aggravating the injury to the patient’s liver function. This resulted in the discontinuation of afatinib. The appraisal opinion believed that the patient’s liver function injury was mainly related to the ADRs of alfatinib, and there was also a certain correlation with the single overdose of ondansetron. Because the patient’s liver function was damaged and alfatinib was a hepatotoxic drug, the treatment of anti-tumor drugs was stopped. It had a slight causal relationship with the development of the patient’s disease to death, and a $10,587 of compensation was assumed by the healthcare services.

The compensation reasons for two cases of DILI were not completely identical. In Case 10, the DILI was not discovered in a timely manner, leading to increased liver function injury. The healthcare services undertook DILI treatment related compensation. In Case 11, the patient suffered from tumor-targeted-treatment interruption due to DILI and died, and the healthcare services also undertook death-related compensation. In the published judgment document, although DILI was identified, the evaluation method was not published. We used RUCAM to reevaluate this case. Case 10 was unable to be re-evaluated with RUCAM because liver function data was not published in the court documents. Current pharmacotherapy options of DILI remain under discussion, but the use of the offending drug must be stopped as soon as DILI is suspected [46]. Therefore, it is important to clarify the DILI diagnosis. RUCAM is the most used causality assessment tool worldwide for the diagnosis of DILI and herb-induced liver injury (HILI) in many epidemiological studies, case reports, and case series [47]. Therefore, it is recommended that, for cases involving DILI, both medical and judicial expertise institutions use RUCAM scoring to: 1) avoid not detecting DILI in a timely manner, which may lead to increased liver function injury, or 2) avoid incorrectly evaluating DILI leading to unnecessary discontinuation of drug.

Although we only reported the liver injury of rituximab, imatinib, sorafenib and alfatinib, which are four NADs in our study, the hepatotoxicity of immune checkpoint inhibitors (ICIs) should also be considered by users [48]. In recent years, ICIs, including programmed cell death protein-1 (PD-1) and its ligand (PD-L1) inhibitors, cytotoxic T lymphocyte associated antigen-4 (CTLA-4) inhibitors, were approved for the treatment of various malignities [49]. ICIs have an immunotoxicity to various organ systems, that is, immune-related adverse events (irAEs), which has also become an unavoidable new complication in clinical practice. ICIs-related irAEs, especially high-grade irAEs, pose a significant threat to patients’ lives, with an incidence ranging from 54% to 76% [50]. One important side effect of all three classes of ICIs is DILI, and they also increase the risk of idiosyncratic DILI of co-administered drugs. Its occurrence often leads to the discontinuation of therapy and might require treatment [51,52]. Immune tolerance plays a dominant role in the immune response of the liver, and impairment of immune tolerance with ICIs increases DILI in both humans and other animals [52]. Therefore, it is necessary for medical service providers to familiarize themselves with diagnostic steps and management plans for patients with liver injury. When assessing for possible ICIs induced hepatotoxicity, it is of utmost importance to use a formal scoring system such as the RUCAM to assess for risk factors, alternative causes, and response to cessation and re-exposure of a given drug [53]. It is hoped that the use of RUCAM will further increase confidence in the studies performed for DILI detection with new drugs, herbs, and dietary supplements, and in the identification of risk factors and new biomarkers to be able to take the appropriate measures to reduce the risk of hepatotoxicity [54].

#### Toripalimab

*Lung injury from irAEs*. Case 12. A male patient over 75 years old with esophageal cancer and lung cancer after surgery developed lung injury and other irAE side effects on the sixth day after treatment with teriprizumab and died after resuscitation attempts. It was identified that the medical service providers had some defects in the process of diagnosis and treatment, such as insufficient communication, attention, observation, and monitoring of the patient’s condition. According to literature reports, there is an overall incidence of 4.5% for ICI-associated pneumonia, and the incidence of severe pneumonia (grade 3 or higher) was 0.8–1.5% [50]. Chen Y et al. [50] reported two Chinese patients with advanced tumors suffering from severe multisystem irAEs. Both patients developed myocarditis and one patient was also diagnosed with organizing pneumonia after receiving toripalimab treatment. They proposed that improved awareness and an early identification of multisystem irAEs are of great importance in management of patients undergoing treatment of ICIs. In addition, we suggest that medical service providers should also improve communication and notification of related risks.

#### Nivolumab

*Adverse cardiac reactions from irAEs*. Case 13. A 40-year-old male patient with lung cancer was not carefully monitored for adverse cardiac reactions during treatment with nivolumab. Specifically, the myocardial zymogram was not rechecked after two sinus tachycardia. Immune-associated myocarditis is the most serious ADRs among all organs with immunotoxicity, and the fatality rate may be as high as 50% [55]. Moslehi’s [56] study on 101 cases of severe myocarditis showed that dosing information was available in 59 patients; 64% received only one to two doses before myocarditis onset. The precise timing of myocarditis onset in relation to ICI initiation was available in 33 patients. Of these, the median onset was 27 days (range 5–155 days) with 76% occurring in the first six weeks. Further, concurrent severe irAEs occurred in 42% of patients, most commonly myositis (25%) and myasthenia gravis (11%). Among all cases, death occurred in 46 (46%). Fatality rates were higher with a combination of anti-PD-1/PD-L1 plus anti-CTLA-4 than with anti-PD-1/PD-L1 monotherapy (67% vs. 36%, p = 0.008). Deaths also occurred in three of five patients with ipilimumab monotherapy associated myocarditis.

Nivolumab, as a PD-1 inhibitor, should be investigated for its irAEs, such as: 1) Before and during the administration of drugs, perform cardiac related examinations, which include cardiac biomarkers and ECG 2) While monitoring and observation some irAEs prodrome symptoms, including dyspnea, myalgia, fatigue, blepharoptosis, and myasthenia; 3) The induction period of irAEs is different so management of the entire process is essential, and any abnormality findings should be addressed quickly. For details, please refer to the ‘Management of immunotherapy-related toxicities, Version 1.2022 [57].

#### Bortezomib

Five cases related to bortezomib were all diagnosed as multiple myeloma, which were associated with ADRs. ADRs occurred during the first course of treatment with bortezomib, among which Cases 14, 15, and 18 occurred after the second (day four) drug treatment. Complications involving: 1) Transient acute ADRs (including blood pressure decline, thrombocytopenia), and peripheral neuropathy; 2) Acute lung injury, including severe pneumonia, dyspnea, ARDS; 3) Cardiac failure. Among these cases, four patients died while one case resulted in grade three disability. Among the death cases, the autopsy confirmed that the cause of death of one patient was consistent with multiple myeloma complicated with infection, secondary drug-induced diffuse alveolar injury of both lungs, and multiple organ failure caused by DIC.

Bortezomib is an important part of current anti-myeloma therapy with a good clinical efficacy and manageable side effects [58]. However, the characteristics of bortezomib-related severe AEs need to be monitored. A comprehensive analysis from the Japanese Adverse Drug Event Report database showed that 13 of the 26 bortezomib AEs extracted presented AE signals. The post-exposure outcomes of 12 AEs showed fatal outcomes at rates exceeding 10%, including cardiac failure (30%), lung disorder (24%), pneumonia (18%), and tumor lysis syndrome (TLS) (10%). Furthermore, a histogram of time to onset revealed that the 12 AEs were concentrated from the beginning to approximately 1 month after bortezomib administration. The median onset times for cardiac failure, lung disorder, pneumonia, and TLS were 28, 13, 42, and 5 days, respectively [59]. These AEs had a higher rate of fatal clinical outcomes after onset than other AEs and exhibited a greater onset tendency in the early post-dose period. Domestic literature analysis [60,61] on ADRs caused by bortezomib showed that the first two organs involved in ADRs are respiratory system damage (28.89–33.3%), followed by central and peripheral nervous system damage (13.33–13.33%). The mortality rate is 19.51–25.81%, among which the mortality rate of lung injury is as high as 50%. A total of 53.23% of the ADRs occurred in the first course of chemotherapy. The remaining ADRs occurred in the second to eighth courses with 3.23% occurred on the day of chemotherapy, and the rest occurred on or after the next day. Lung injury occurred in the first 3 weeks [60,61]. A Japanese study [62] reported that before bortezomib approval in Japan, four (30.7%) of 13 patients with myeloma who were treated with privately imported bortezomib experienced fatal pulmonary complications. Of these four patients, two (15.4%) died from respiratory failure. Another study [63] reported that of the 1,010 patients registered, 45 (4.5%) developed lung disease, five (0.50%) of whom had fatal cases. The median time to lung disease onset from the first bortezomib dose was 14.5 days, and most of the patients responded well to corticosteroid therapy. In some cases [64], a pre-existing cardiac disease was an associated risk factor for further bortezomib-related cardiac disorder, while another case report highlighted a patient with no previously known cardiac disorder or any cardiovascular risk factor who suffered from bortezomib-related heart failure.

Therefore, combined with medical errors and literature, we suggest that: 1) Systematic cardiac and lung screening should be performed before and during administration of bortezomib; 2) There is a need to monitor signs of cardiac failure, lung disorder, and pneumonia, potentially resulting in serious outcomes; 3) The usage and dosage of bortezomib should be standardized to reduce the risk of medication; 4) Focus on high-risk groups prone to ADRs and appropriately extend the stay for observation, and 5) Fully explain the benefits and risks of medication and off-label use, and obtain informed consent.

#### Apatinib

Both patients with lung cancer (Cases 19 and 20) died of massive hemoptysis, and two patients with gastric cancer (Case 21) and cervical cancer (Case 22) died of massive gastrointestinal bleeding.

*Massive hemoptysis*. Hemorrhage or abnormal clotting time is one of the main side effects of anti-angiogenic drugs, and it often occurs within 1 month after taking the drugs. Wang W et al. [65] reported that two patients with advanced esophageal cancer developed fatal hemoptysis (grade 5) after treatment with apatinib. During the treatment, both patients showed an abnormal coagulation function, APTT of one patient was slightly prolonged after 3 weeks of treatment using apatinib orally, and another patient showed a significant increase in PT and APTT after 2 weeks of treatment. However, no obvious abnormality was found in liver and kidney function except for the low plasma protein. It was suggested that the abnormal blood coagulation function should be associated with the side effects of apatinib. Further, the treatment with apatinib caused tumor necrosis, and the tumor continued to progress and invaded the bronchial artery, leading to hemoptysis by rupture of vessels [65]. Lung cancer is a cause of hemoptysis, and central lung squamous cell carcinoma (LSCC) is prone to cavity formation and sudden fatal hemoptysis. Apatinib may induce or aggravate hemoptysis [66]. Yang D et al. [67] reported that apatinib treatment was used for three patients with advanced non-small cell lung cancer (two cases of adenocarcinoma and one case of squamous cell carcinoma). The squamous cell carcinoma case died of sudden hemoptysis (grade 5). Although they have no definite conclusion regarding the causality between hemoptysis and apatinib treatment, a potentially increased risk of squamous cell lung cancer should be considered when using apatinib in non-small cell lung cancer (NSCLC) patients. The risk of hemoptysis in squamous NSCLC patients is inconclusive and current limited data suggest that squamous NSCLC may not be a contraindication of apatinib. A single-arm, open-label, investigator-initiated phase II prospective study demonstrated that the incidence of hemoptysis treated with low-dosage apatinib monotherapy in advanced LSCC was 18.4% (n = 38, grade 1 or 2) [68]. However, another phase II single-arm trial study revealed that the incidence of hemoptysis treated with apatinib as a maintenance therapy following standard first-line chemotherapy in extensive disease small cell lung cancer (SCLC) was 8.3% (n = 12, grade 1–2) [69]. Zeng DX et al. [70] reported that four advanced lung adenocarcinoma patients with a KRAS mutation were orally administered apatinib (250 mg/d) after second-line treatment, and one patient developed manageable hoarseness and hemoptysis (grade 1).

*Gastrointestinal bleeding*. Xu Z et al. [71] reported that four patients with a clinical diagnosis of stage IV gastric adenocarcinoma were treated with apatinib (500 mg/d) combined with paclitaxel and 5-fluorouracil, and one patient had gastrointestinal bleeding (grade 1). Li X et al. [72] reported that microwave ablation combined with apatinib and carbamazumab was treated in patients with advanced hepatocellular carcinoma and the incidence of gastrointestinal bleeding (grade 3 or 4) was 14.3% (n = 14, grade 3–4). Li XF et al. [73] reported that a 55-year-old Chinese woman with advanced gastric cancer suffered from gastrointestinal bleeding and perforation on the nineteenth day of apatinib administration. Considering the patient’s disease and history of drug use, the author thought that the most possible causes of patient’s upper gastrointestinal bleeding and perforation included drug resistance and cancer progression, in combination with adverse effects of apatinib.

In summary, apatinib has the potential to increase the risk of bleeding, therefore, we have learned that apatinib should be administrated with caution. To address these risks: 1) Hemorrhage and hemoptysis are both emergencies that need immediate treatment. Therefore, it is necessary to establish and prepare corresponding rescue procedures before using these drugs. 2) The blood clotting function should be monitored regularly. Patients on anticoagulant therapy combined with warfarin should be routinely monitored for prothrombin time (APTT) and internationalized normalized ratio (INR). Bleeding should be closely monitored when using the drug, especially when administered to patients undergoing additional treatment. Close attention should be paid to clinical signs of bleeding. The patients with hematemesis, hemoptysis, or black stool should reduce their dosage or stop the medicine. Additionally, for patients with severe bleeding (grade 3 or 4), it is recommended to temporarily stop the use of apatinib. If a severe hemorrhage reoccurs after the medication is resumed, the drug can be continued after the dose is lowered. If the adverse reaction persists, it is recommended to stop the drug [74]. 3) Special attention should be paid to the possibility of massive bleeding caused by tumor erosion and necrosis. Physicians should focus on choosing suitable candidates, detailed assessment, close surveillance, and active management to minimize lethal cancer-related complications besides yielding ideal tumor control [75]. 4) Physicians should use drugs according to the indications of the drugs. In the event of off label use of apatinib, communication should occur, as well as notification of drug risks in all links.

#### Bevacizumab

*Gastrointestinal bleeding*. Case 23. A 56-year-old female patient with glioma was given bevacizumab intravenously while blood pressure was not controlled within the normal range. It was determined that the timing of administration was improper, and the indications were insufficient, which led to gastrointestinal bleeding, hematemesis, airway obstruction, and asphyxia.

Case 24. A patient with primary liver cancer had a history of gastrointestinal hemorrhage, and the doctor failed to fully explain the reasonable off-label use of drugs under special circumstances, which was a contributing factor in the death of the patient along with hemorrhagic shock caused by drug-induced gastrointestinal bleeding. The most serious adverse effects of bevacizumab are gastrointestinal perforations, surgery and wound healing complications, and hemorrhage [76]. A study in France showed that the most common adverse reactions of bevacizumab involved the gastrointestinal system (21.9%), in which hypertension and gastrointestinal hemorrhage accounted for 2.7% and 2.7%, respectively [77]. Therefore, all patients on bevacizumab treatment are recommended to have blood pressure monitored every two to three weeks.

#### Crizotinib

An increased risk of PE in patients receiving crizotinib treatment was also reported in clinical trials but was easily neglected due to relatively low incidence [78].

*Pulmonary embolism (PE)*. Case 26. A patient with lung cancer using crizotinib had a PE during treatment. It was identified that the medical service provider did not give corresponding information about the potential risks of the drug, and there was no communication record of special drugs. The patient possessed multiple risk factors, including advanced age and reduced mobility, which increased the risk of pulmonary embolism for crizotinib. Therefore, communication and risk notification should be strengthened for high-risk and other special groups.

#### Errors in off-label drug use

Outside of direct medical damage caused by ADRs, medical errors can also cause indirect medical damage. Off-label drug use is particularly common for treatment of malignancies due to the existence of numerous cancer subtypes, difficulties involved in performing clinical trials, rapid diffusion of preliminary results, and delays in approval of new drugs by regulatory bodies [79,80]. Off-label drug use refers to prescribing medicines in a manner that is inconsistent with prescribing information published by regulatory authorities. Off-label drug use can be classified into different categories which include unapproved indication, use in a special population, an unapproved route of administration, or with a dose not specified on the FDA-approved label [81]. In our study, errors in off-label drug use ranked second among medical technology errors, with the primary errors being errors in the management of off-label use.

Case 14. Unapproved dose involved an overdose of bortezomib chemotherapy. A 67-year-old patient with multiple myeloma developed symptoms of hypoxia soon after using bortezomib with a dose approximately 1.46 times that of the recommended dose in the manual, and the hypoxia continued to progress. It was identified that the medical service providers did not anticipate the possible drug toxicity after the overdose of bortezomib, and the patient’s respiratory symptoms were not identified in time. This led to a missed diagnosis and the failure of timely and effective treatment of patients with ALI / ARDS, resulting in further development and deterioration of the patient’s condition. As a result, it was decided that the medical service providers shall bear 65% of the liability for compensation.

Case 17. A case from 2017 of unapproved route of administration involved a patient who died suddenly after treatment with bortezomib for multiple myeloma. It was confirmed that the patient’s death was caused by a combination of the original disease and from possible heart failure caused by a drug-induced injury. The doctor used an unapproved route of administration, injecting the drug subcutaneously, rather than intravenously, per instructions. While there was no causal relationship with the death, it was considering medical errors. The appraisal and court required the hospital to assume 20% of the liability for compensation. In 2017, there was sufficient evidence for the subcutaneous administration of bortezomib. The study found that subcutaneous bortezomib offered non-inferior efficacy to standard intravenous administration, with an improved safety profile [82] and alternative subcutaneous administration should be considered instead of intravenous administration in use of bortezomib for patients with multiple myeloma [83]. However, the court and appraisal opinion held that the medical service providers had not fulfilled the corresponding procedures for off-label use resulting in the 20% assignment of responsibility.

Unapproved indication included: a) apatinib (unapproved for: cervical cancer, early-stage gastric cancer, lung squamous cell carcinoma), b) bevacizumab (liver cancer, brain glioma, diffuse astrocytoma), c) gefitinib (undifferentiated large cell lung cancer), d) pezopanib (carcinoma of renal pelvis), and e) trastuzumab (breast cancer).

Case 28. A patient with renal pelvis cancer with multiple metastases was treated with pezopanib, and it was identified that the medical behavior of the healthcare services did not violate diagnostic and therapeutic norms. However, the healthcare services did not comply with the corresponding procedures and fully inform the obligation to use the drug in accordance with off-label use of NADs. Based on later examinations, it was demonstrated that the use of this drug had a certain effect on alleviating the patient’s condition. The patient had renal pelvis cancer with multiple metastases, with poor prognosis, and a lack of clear targeted drugs for treatment. There is no significant causal relationship between the outcome and the diagnosis and treatment behavior of the healthcare services, but it is caused by the patient’s own illness. There is a causal relationship between the medical errors behavior and the medical expenses incurred by the patient using pezopanib. It is recommended that the magnitude of the causal force be the main reason.

The *doctor law of the people’s Republic of China* [84], which came into force on March 1, 2022, wrote medication off-label use into the law for the first time. According to Article 29 of the law, "1) doctors should adhere to the principle of safe, effective, economical and reasonable drug use; 2) follow the guiding principles of clinical application of drugs, clinical diagnosis and treatment guidelines, drug instructions and other rational drug use; 3) in the absence of effective or better treatment methods and other special circumstances; 4) after doctors obtain the clear informed consent of patients; 5) the use of drugs not specified in the drug instructions but with evidence-based medical evidence can be used for treatment. It should be noted that the GPCANADs also propose that medical service providers should formulate corresponding management systems and technical specifications to strictly manage the use of drugs that are not specified in the drug instructions but have data to support evidence-based medicine. Under special circumstances, the right to use NADs should be limited to doctors with senior professional and technical titles authorized by the tertiary hospital, and drug use monitoring and tracking observation should also be performed [8]. Pharmacists should examine the suitability of doctors’ prescriptions and medication orders and strictly regulate doctors’ medication behavior. Taking the judgment of cases of off-label use into consideration, when assessing NADs for off-label use, medical service providers should maintain usage in accordance with the provisions of the national laws and regulations to avoid medical disputes caused by such errors.

#### Errors in condition evaluation during diagnosis and treatment

There was one case of MAM (Case 29) and three cases of II (Cases 30, 31, and 33) caused by errors in condition evaluation.

Case 29. The doctor mistakenly diagnosed the patient as suffering from pancreatitis and stopped using chidamide, leading to the deterioration of lymphoma, which was identified as having a causal relationship with death.

Case 30. A patient with post-operative metastasis of undifferentiated large cell carcinoma of the (right) lung (upper lobe) received targeted drug therapy with gefitinib. It was identified that the monitoring and evaluation of the efficacy of patients during diagnosis and treatment, as well as the treatment of ADRs, are not timely. There is a lack of scientific and accurate relevant records.

Case 31. A patient with adenocarcinoma of the right lung who had previously failed to use gefitinib for treatment outside the hospital, and subsequently switched to other chemotherapy regimens, but was re-treated with gefitinib without a systematic assessment of the patient’s condition by the healthcare services. It was identified that the healthcare service examination before treatment was incomplete, the evaluation of medication indications was insufficient, and the notification was insufficient. There was an improper treatment, which had a causal relationship with the patient’s death.

Case 33. A patient with undifferentiated carcinoma of the nasal sinuses and nasal cavity was identified as having a high possibility of death due to bilateral lung metastasis, bone marrow suppression, secondary lung infection, and ultimately respiratory and circulatory failure after treatment. The main errors of the healthcare services are: 1) For the treatment of advanced head and neck tumors, there is no multidisciplinary consultation to jointly determine the treatment plan. As radiotherapy is a commonly used treatment method for head and neck tumors, the healthcare services should also request the radiotherapy department to consult, determine the treatment plan, inform the patient of the different plans and risks, and seek the patient’s opinions before treatment. There are shortcomings in the healthcare service diagnosis and treatment and nimotuzumab was given without informing the radiotherapy plan. 2) The healthcare services are at fault for not fully informing the patient’s condition, treatment plan, and prognosis. 3) The doctor did not perform gene testing before treatment, and the paraffin section tissue test showed normal expression levels. The doctor made a mistake in using the drug.

Therefore, in the diagnosis and treatment process, timely monitoring and evaluation of the efficacy of patients treated with NADs should be carried out, and relevant records should be scientifically and accurately kept. When reusing drugs that have failed treatment, the patient’s condition and indications for NADs should be fully reassessed. For the treatment of advanced head and neck tumors, and the need to interrupt the treatment of NADs, it is recommended to negotiate and jointly determine the treatment plan among medical professions in multiple disciplines.

#### Errors in molecular target detection

There were two cases of II (Cases 27 and 32) and two cases of MAM (Cases 9 and 35) caused by errors in molecular target detection.

Case 27. A patient with primary bronchogenic lung cancer (stage IV of left upper central type high to moderately differentiated adenocarcinoma) was treated with crizotinib when the ALK gene deletion mutation was shown. It was identified that the ALK gene deletion mutation was not the result of driving gene fusion mutation. In light of the positive ALK results, the patient has used the targeted drug crizotinib for nearly one year, even no further detection and confirmation efforts were made. There was an incomplete analysis of the medical condition during the diagnosis and treatment process. The errors included unclear medication indications, incomplete handling opinions, or incomplete medical advice.

Case 32. A patient with stage IV right lung adenocarcinoma was advised to take gefitinib without genetic testing, which was inappropriate. It was identified that there was no direct causal relationship with the patient’s death, but it could be considered as a minor cause of death.

Case 9. When the results of pathological and gene mutation tests are not available, a patient with low-grade malignant potential mesenchymal tumor was advised to take imatinib orally and consider surgical treatment after the tumor shrank. One month later, the c-KIT/PDGFRa gene mutation test results showed that the c-KIT gene in the sample was of wild type in exons 11, 9, 13, and 17, and the PDGFRa gene was of wild type in exons 12 and 18. Compared with previous MRI scans, the tumor was slightly plump and enlarged. The appraisal and court held that the medical authorities had delayed further treatment of the tumor.

Case 35. A patient with breast cancer (cT1N0M0 and HER2 negative) was wrongly recorded as positive, which led to the incorrect use of trastuzumab. The appraisal and court held that there was a causal relationship between the use of the drug and the costs incurred.

Therefore, for drugs with clear targets of action, the principle of target detection must be followed before use. Attention should also be paid to the relevant detection methods and the correct interpretation of the detection results. Before prescribing NADs for target detection, it is necessary to review and clarify the target detection report again to avoid errors in this process.

#### Errors in histopathological diagnosis

There were four cases of MAM caused by errors in histopathological diagnosis.

Case 34. A patient with nasopharyngeal carcinoma was misdiagnosed as having a recurrence in the parotid region after radiotherapy and chemotherapy, and the postoperative pathology suggested that "no tumor was found". The appraisal and court judgment identified that there was a causal relationship with the patient’s repeated treatment with three cycles of nimotuzumab.

Case 37. A 42-year-old female patient presented with a left lung mass accompanied by mediastinal lymph node enlargement, as indicated by a plain CT scan and enhancement. Physical examination revealed that an enlarged lymph node about 2 × 1 cm in size could be palpated on the left clavicle. Biopsy and cytological examination of the lymph node revealed metastatic poorly differentiated lymph nodes (adenocarcinoma). Therefore, left lung cancer with mediastinal and supraclavicular lymph node metastasis (CT2N3M0 stage IIIB) was diagnosed, and radiotherapy, chemotherapy, and erlotinib targeted treatment were given. Three years and seven months later, due to the discovery of enlarged left axillary lymph nodes, Hodgkin’s lymphoma (mixed cell partial nodular sclerosis) was diagnosed after biopsy and immunohistochemical examination. The appraisal and court held that cytological diagnosis was not sufficient to determine clinical diagnosis, and the medical team did not conduct further tumor histopathological examination to confirm the diagnosis. In the absence of diagnosis of lung adenocarcinoma and target testing to determine whether EGFR was positive, oral erlotinib treatment was received. These errors led to an extension of the patient’s treatment cycle in the later stage, as well as more uncertain factors in the treatment in the later stage. The appraisal held that the healthcare services should carry 40% of the responsibility. However, the court ruled that the healthcare services should bear 100% of the responsibility. The disability appraisal was not carried out at the time of the judgment, and the patient reserved the right to further litigation. This case resulted in the largest compensation settlement of all 39 cases at $97,301.

Case 38. A patient with invasive ductal carcinoma of the left breast was not treated with endocrine and targeted anticancer therapy. The appraisal and court held that the lack of comprehensiveness and accuracy of immunohistochemical detection, pathological molecular typing diagnosis, and a misdiagnosed of triple negative breast cancer, led to incomplete treatment plans. At the time, it was not determined that the patient’s breast cancer was a hormone dependent tumor with a high expression of estrogen receptor (ER). Endocrine therapy was not given, and targeted anti-cancer treatment was lacking. There was a certain causal relationship between inhibition, reduction, and avoidance of tumor recurrence and metastasis, or the effect of prolonging the patient’s survival was ultimately affected.

Case 39. Without pathohistological diagnosis, Fanconi Syndrome (after treatment with adefovir dipivoxil for hepatitis B) was misdiagnosed as bone metastasis of liver cancer, and sintilimab targeted therapy was wrongly used. The appraisal and court held that there was a slight to secondary causal relationship with related cost losses.

The lessons learned from the study support following these principles: 1) Treatment can be used only after histopathological diagnosis, 2) Drugs with clear target can only be used after target detection, and 3) Indications must be strictly followed.

#### Inappropriate treatment of NADs

There were three cases of DDAT caused by improper timing of the administration of NADs and delayed treatment.

Case 7. A patient with Philadelphia chromosome positive acute lymphocytic leukemia (Ph + ALL) did not consider timely addition of TKI drugs such as imatinib.

Case 8. A patient with gastrointestinal stromal tumor was not informed to take imatinib after surgery to reduce chance of recurrence.

Case 36. A patient with infiltrating ductal carcinoma (right breast) was treated with trastuzumab only after finding cervical lymph nodes and liver metastases due to cost reasons. The appraisal and court judgment ruling found that incomplete chemotherapy, especially the delayed treatment of trastuzumab, and the failure to fully fulfill the obligation to inform during the treatment period, had a causal relationship with metastasis, recurrence, and death.

Therefore, during the treatment process, medical service providers need to understand the timing of treatment for NADs.

### Medical ethics errors

Medical ethics errors were mainly caused by the failure of medical personnel to fulfill their obligations of explanation and disclosure, which infringed on the informed consent rights of patients, accounting for 24.26% of this study. Seven categories from our study are highlighted (Table 5). Among them, there were more errors in informing in three aspects: 1) Possible ADRs, risks, and prognosis; 2) Detailed introduction to alternatives treatment; and 3) Risks and benefits of off-label drug use. In addition, we also found an inadequacy in medical informed consent when changing treatment schemes, when restarting medications that had previously failed treatment, in performing an autopsy after death, and additional risks in special circumstances. Inadequate notification provided above may affect patients’ ability to choose treatment schemes and violate the patients’ rights in choosing better options for informed consent. Therefore, medical service providers need to explain and communicate to patients throughout the process.

### Medical record writing / safekeeping errors

Medical records are essential resources for evaluating a patient’s medical history, progress, and care. They are the most original and objective written records that demonstrate the entire process of medical behavior from occurrence to completion. Medical records also constitute legal documents that can serve as evidence for medical appraisal and determination of facts by the courts [21].

Case 10. The patient did not have relevant outpatient medical records written by healthcare services according to the regulations during the three outpatient visits. The appraisal and court judgment found that there was no evidence to prove that the healthcare services had fulfilled their obligations to inform the patient of medication precautions and monitor liver function.

Case 30. Due to the lack of scientific and accurate records of efficacy monitoring and the evaluation of ADRs in the process of diagnosis and treatment, it is believed that this impacted the ability to clarify the treatment effect and formulate the next treatment plan. The writing of medical records is not standardized and incomplete, and the healthcare services may be deemed to have failed to fulfill the full obligation of disclosure and caution.

Case 31. The patient had recorded chest tightness, increased suffocation, and intermittent fever in another hospital. However, when they arrived at the defendant’s hospital, the change was not mentioned in the medical record, and the physical examination records showed that the general condition of the patient was acceptable, with no significant abnormal physical signs. The appraisal and court judgment ruling found that the doctor was not diligent in assessing the changes in the patient’s condition.

Case 35. A patient with breast cancer (cT1N0M0 and HER2 negative) was wrongly recorded as positive in the progress note, which led to the incorrect use of trastuzumab. It was identified that there was a causal relationship between the use of the drug and the costs incurred.

Therefore, doctors should pay attention to the accuracy, timeliness, and completeness of medical records.

### Risk prevention and control strategies

Based on the finding of our study and supportive studies, medical service providers should consider the strict implementation of the management norms for the clinical application of NADs, follow the basic principles of the clinical application of NADs, and heed the warnings on drug labels and have a comprehensive understanding of the ADRs associated with NADs. Special attention should be paid to acute IRR, bleeding, and irAEs (including HBVr, cardiotoxicity, lung injury, liver injury, and infection), and before using drugs, medical service providers should make clear whether patients should avoid, stop, or suspend the use of the drugs when they have comorbidities or complications or when related conditions occur. Patients should be monitored for the precursor symptoms of ADRs and established emergency rescue systems should be in place to respond to ADRs. In addition, the medical community should strengthen the management of off-label use of NADs, the communication and informed consent of patients, and the writing of medical records. Based on the litigation cases that have been discussed and clinical experience, we suggest a framework that may be helpful to prevent and control risk for NADs use (Table 6).

**Table 6:** Strategies for risk prevention and control for NAD-related disputes.

Order number	Recommendations
**Before**	
**1**	**Strictly abide by NAD-related health laws, regulations, rules, and diagnostic- and treatment-related norms and routines, and abide by professional ethics**
①	Determine whether the malignant tumor is confirmed by histological or cytological pathology or confirmed by special molecular pathology. Special cases require multidisciplinary consultation.
②	For drugs with clear targets, target must be detected before use.
③	Follow the instructions of package insert strictly.
④	Perform rational use of drugs under special circumstances. Strictly follow the management plan for off-label use and obtain informed consent.
⑤	Pay attention to the selection of treatment plans, provide patients with written information about the proposed treatment plan and possible alternatives plans, and explain the associated benefits and risks.
**2**	**Pay attention to ADRs**
①	Perform a drug evaluation before using NADs. It is necessary to read the drug manual in detail, consult relevant literature, pay special attention to serious ADRs, and contraindications of drugs. Identify the population or situation where the drug is not suitable for use. Improve NAD-related ADR risk identification, assessment, and prevention and control measures, prepare response plans, especially the rescue mechanism of serious ADRs, and take the initiative to prevent risks.
②	Evaluate the patient’s condition and improve necessary relevant examinations and tests.
③	Evaluate the medication and allergy history of patients and NADs interaction with the other drugs. Determine the plan of inspection and tests that need to be monitored at each stage before, during and, after drug use and whether to stop or adjust the dose. Develop a serious ADR pharmaceutical care plan.
④	Obtain informed consent with detailed information of medication about the risk of possible ADRs. Patients should be informed of three special situations: the unique situation of the patient, the risks regarding the usage of the drug, and precautions that will be taken.
⑤	Educate patients on medication, improve their medication compliance, and self-protection awareness to find early signs of ADR as soon as possible and intervene.
**During**	
**1**	**Fill in and keep medical records properly**
①	The writing of medical records shall follow the principles of objectivity, truthfulness, accuracy, timeliness, integrity, and standardization.
②	The rescue record shall be completed within 6 hours after a rescue and the rescue process needs to be specific.
③	A person shall be appointed to check whether the medical records are complete and keep them properly to prevent loss.
2	**Establish and improve doctor-patient communication; patiently investigate, explain, communicate, and explain the consultation, opinions, suggestions, and questions raised by patients during diagnosis and treatment.**
3	**Closely observe and monitor ADRs and strengthen pharmaceutical care.**
①	Timely reexamination relevant inspection and test indicators.
②	Patrol inspection shall be strengthened during drug administration to deal with any change in the condition in a timely manner.
③	The prodrome regarding ADRs will be followed strictly, and vital signs will be monitored closely.
4	**Immediate active and proper management for the post-medication ADRs.**
①	If an ADR occurs, it should be handled and treated immediately to reduce or mitigate the patient’s drug-induced injury.
②	Communicate with the patient or their guardians.
③	Record the patient’s ADR situation and treatment measures in a timely fashion.
④	If an ADR occurs during infusion, both doctors and patients shall seal or unseal the infusion on site jointly.
⑤	Re-educate patients and their guardians about potential ADRs.
**After**	
1	**Strengthen monitoring after medication, pay attention to delayed ADRs.**
①	Drugs requiring close observation after administration shall be observed at the time specified in the package insert.
②	It should be noted that some drugs need to be observed for a period before the infusion of other drugs, i.e., it is recommended to observe a patient for 30 to 60 minutes after the infusion of pertuzumab before giving follow-up trastuzumab or chemotherapy.
③	Inform patient about the symptoms of delayed ADRs, guide patient to observe and monitor after discharge, and instruct the patient regarding emergency disposal measures after the prodromal symptoms of delayed ADRs.
④	Explain to the patient in detail about the indicators that need to be reexamined and precautions that should be taken after discharge.
⑤	Clinicians, nursing staff, and clinical pharmacists should closely follow up on the drug-related toxicity of patients and report ADRs in a timely fashion, especially serious and newly discovered ADRs.
2	**If serious ADRs occur, consult with hospital lawyers on how to prevent medical disputes. If the patient dies, inform close relatives of the autopsy regulations.**
3	**Clinical search for alternative causes.**
①	Organize treatment departments and hospital experts to discuss and comprehensively analyze medical disputes and professional behaviors and find out alternative causes for patient’s comorbidity and disease factors.
②	Organize death case discussion to find other cause of death.
4	**Regularly analyze and report the dynamics and trends of NADs, as well as the standardization of diagnosis and treatment behaviors. Carry out meetings of the health department of the adverse event, to understand the medical errors in MMLD, learn from experience, and avoid same mistakes.**
5	**Implement medical quality and safety management systems, organize medical quality and safety assessments, analyze medical quality and safety information, and formulate preventive measures against the risks found.**
6	**The healthcare facility should undertake to ensure and verify the professional training about its healthcare professionals and establish corresponding management systems and technical specifications to strictly manage and standardize the use of NADs.**

## Conclusion

Our study investigates the MMLD litigation related to NADs, analyzes its general characteristics, causative drugs, resulting injuries and medical errors, and gives examples of typical cases. Based on our study, we put forward risk prevention and control strategies for NADs. Through case studies, the intent was to support clinicians in further improving their understanding of NAD-related litigation, learn about common medical errors related to NADs, and take preventive strategies to reduce MMLDs. Both doctors and patients may make better choices to resolve MMLDs through consultation, which can save time and litigation costs. The number of NAD litigation cases has increased rapidly in the past six years, which is related to the improvement of the accessibility of NADs in China and the clinical application of additional, new NADs. Therefore, it is necessary to raise awareness of the rational use of NADs and strengthen management. The autopsy rate is low and medical service providers should discuss fatal cases and find alternative causes in support of the physician.

We also found that the most common damages were closely related to death, disability, and increased expenditure on treatment caused by ADRs, II, DDAT, and MAM. The most common NADs involved in medical errors were rituximab, bortezomib, apatinib, and imatinib. We need to pay attention to irAEs related to ICIs and other serious ADRs of NADs and prepare rescue and treatment measures in advance. It is necessary to implement a comprehensive management process for addressing ADRs. Improving patients’ awareness of ADRs and self-management, and monitoring, observation, and management of ADRs are effective prevention strategies to reduce and avoid MMLD. We need to determine the observation and monitoring plan of patients before, during and after drug use, and identify the specific inspections and test items for monitoring. The most common medical errors include technology errors, ethics errors, and medical record writing/safekeeping errors. Before using a NAD, we need to make clear the histopathological diagnosis and target detection. The risks of NADs and off-label use should be fully communicated to patients, and the management of the off-label use of NADs needs to be strengthened. Finally, management measures such as writing new and keeping existing medical records are also essential.

However, several limitations to this study should be noted. We included only judgments resolved by civil lawsuits. Studies have shown that about 10% of medical disputes are solved by civil lawsuits in China [85–87]. More data from additional sources are needed to conduct a more robust evaluation. Nevertheless, any possible selection bias in our study does not affect the internal validity of our findings. China Judgments Online [20] has been used in multiple malpractice analyses, and our analysis could provide guidance for the implementation of risk prevention measures and control strategies to reduce damages, improve patient safety, and limit medical litigation.

## Supporting information

S1 Table Literature retrieval and study characteristics. (XLSX)

S2 Table Detailed summary of thirty-nine-cases concerning novel antineoplastic drugs. (XLS)
